# Medical Items

**Published:** 1915-11

**Authors:** 


### MEDICAL ITEMS

### PHYSICIANS AND THEIR FAMILIES WHEN VISITING NEW YORK CITY SHOULD REGISTER AT THE HOTEL BRESLIN.

Located in the very heart of the city at 29th Street and Broadway. Five minutes' walk to the New York Post Graduate Medical School and eight minutes, surface car, to the Polyclinic Medical School. Three minutes to the Pennsylvania Station. Three minutes from five of the largest department stores. Six minutes from twenty of the principal theatres. The elevated and subway stations conveniently accessible. Hudson Tubes and Fifth Avenue busses two blocks away.

The BRESLIN is a high class house with the most moderate rates in the City of New York. A real home-like hostelry with every modern comfort and convenience—catering only to the best people. The spacious lobby fitted with luxurious easy chairs is inviting to the tired shopper or sightseer. The quiet air pervading the whole is conducive to a home-like atmosphere found nowhere else in New York City. The culinary department is under the supervision of an expert chef—an artist and past-master in the preparation of good things to eat. The Dixie Room is noted as the best place in New York City to get real OLD FASHIONED SOUTHERN cooking. The BRESLIN serves a DIXIE DINNER for a dollar which cannot be equaled. The service in every department is pleasing and satisfactory. Rooms with running water from $1.00 to $2.00. With bath or shower from $1.50 to $3.00.

Everything new and modern, equipped to satisfy the most exacting taste. Enquiries cheerfully answered by Mr. Roy L. Brown, Manager, Hotel Breslin, New York City.

---

### BLOOD AND ITS DISEASES.

This little epitome is devoted exclusively to the subject of Blood. The author has endeavored to incorporate into this small work everything known on the subject to date which is deemed to be of sufficient importance for the practitioner to know.

Accordingly, a section has been devoted to the discussion on the diagnosis of the diseases of the blood which includes all the forms of anemia, chlorosis, leukemia, etc. The whole treatise is written with especial regard to diagnosis.

An equally interesting section on hematology also appears in the most abridged, concise and comprehensive form which will appeal to the laboratory worker. For instantaneous reference it is second to no other work on the subject.

Furthermore, the medico-legal aspect of this subject is briefly discussed and the most important test for blood stains are presented in the clearest and most simplified manner.

The section devoted to the Wassermann Reaction and its modifications (the Nogouchi and that of Fields') will undoubtedly impress the reader because of its simplicity and unique arrangement. And, not only is the serum-diagnosis of syphilis discussed but also the serum-diagnosis of typhoid fever and that of pregnancy.

Due acknowledgment and credit is hereby given to the many authorities whose works have been consulted during the compilation of this brochure, and because of the limitation in space the bibliography has been omitted.

A copy of this book will be gladly sent to members of the medical profession whenever so requested.

Battle & Co., Chemists Corporation, St. Louis, Mo.

---

### BACTERIAL-VACCINE THERAPY.

The treatment of infectious diseases with preparations derived from corresponding micro-organisms long since passed the experimental stage, and bacterial vaccines may be said to occupy an assured place in therapeutics. These vaccines, as is doubtless well known to most physicians, are suspensions, in physiologic salt solution, of killed bacteria. An important effect of their administration is to raise the destructive power of the patient's leucocytes against the specific living invaders. Injected into the human organism, bacterial vaccines have an effect similar to that produced on the horse by the introduction of toxins or killed cultures: they cause active immunity. In other words, the administration of a dose of bacterial vaccine stimulates the patient to produce an additional supply of antibodies, thus enabling him to resist the disease.

Bacterial vaccines have several advantages over the ordinary forms of medication. They are determinate or specific in the respective infections in which they are indicated. Their employment relieves the patient of the necessity of frequent "dosing." Being administered by the physician, or under his direct supervision, they enable him wholly to control his cases.

Some idea of the scope which bacterial vaccine therapy has come to assume may be gathered from an announcement which Parke, Davis & Co. are making in current medical journals and which physicians will do well to consult. Twenty-three vaccines are listed in the advertisement. They are supplied in 1-Cc. glass syringes, 1-Cc. glass bulbs, 5-Cc. vials and 20-Cc. bottles, all sealed in a manner that guarantees the sterility of their contents. The syringes are designed for the use of physicians who desire to inject the fluid without first removing it from the original container.

Parke, Davis & Co.'s bacterial vaccines are scientifically prepared, and precise therapeutic results may be confidently expected from their administration.

---

Antiphlogistine provides the best, most agreeable and convenient known means of supplying continuous moist heat, in all inflammations. This can be maintained for 24 hours or longer, at a uniform temperature. Ordinary poultices soon become cold, clammy and uncomfortable to the patient and lose any remedial effect they may have had, before becoming cold.

---

### AN EFFICIENT SOMNIFACIENT.

The great disadvantage attaching to most agents used to produce sleep is that the patient suffers from their untoward effects. This does not apply to PASSADYNE (Daniel) in any manner at all, and yet it possesses somnifacient properties, in a marked degree.

PASADYNE (Daniel) may be depended upon to fulfill the purpose for which it is given, and owing to its freedom from disagreeable properties, it is of special volue in the nervousness and insomnia of women.

A sample bottle may be had by addressing the laboratory of John B. Daniel, Inc., Atlanta, Georgia.

---

A Pennsylvania physician reports:—

"For a patient with a swelling of the knee, rheumatic in character and an exaggerated flexion which had existed for five months, I prescribed Tongaline with marked improvement."

